# What happened during the period from senior medical students’ withdrawal of their applications to take the Korean Medical Licensing Examination in August 2020 to their taking the licensing examination in February 2021

**DOI:** 10.3352/jeehp.2022.19.3

**Published:** 2022-01-28

**Authors:** Sun Huh

**Affiliations:** Department of Parasitology and Institute of Medical Education, College of Medicine, Hallym University, Chuncheon, Korea; The Catholic University of Korea, Korea

## The Korean government’s proposal to increase the quota of medical students and establish a new medical school during the COVID-19 pandemic period

After the outbreak of the coronavirus virus disease 2019 (COVID-19) pandemic in January 2020, physicians and nurses have done their best to combat this novel viral disease in Korea. Starting in February 2020, there was a sharp increase in this viral infection in Daegu [1,2]. It was hard work for medical personnel to diagnose and care for the infected patients. They also had to protect themselves from the infection [3]. During the war against this novel coronavirus, on July 23, 2020, at the National Assembly, the Korean government’s Ministry of Health and Welfare and the ruling Democratic Party of Korea announced a plan to establish a new medical school. It aimed to train physicians who would work in the public sector for at least 10 years after graduation [4]. The specific plans were as follows:

- There will be an increase in the number of medical students by 4,000 over the next 10 years, and the medical students’ quota will be returned to its current state in 2032.
- The current capacity of 3,068 medical students per year will be increased by 400 starting in 2022 to 3,458 to resolve the shortage of medical personnel in provinces and the imbalance across specialties.
- A total of 4,000 additional medical students will be trained for 10 years, 400 each year. Of the 4,000, 3,000 are obligated to serve in essential medical care in the provinces for 10 years.
- In addition, by February 2021, a medical school quota screening for each university will be allocated, and new medical schools will be actively promoted in provinces without medical schools. In 2024, the National University of Public Health and Medicine will open.

## Resistance of the Korean Medical Association, residents, and medical students to the government’s proposal and resolution

However, the Korea Medical Association (KMA) officially objected to the government’s plan for the following reasons [5]:

“Even if physicians are trained through the establishment of a public medical school, it is evident that the purpose of expanding public health care cannot be achieved unless the current public health system is improved and collaborative relationships with private medical institutions are established as prerequisites. In addition, Korea does not lack physicians in absolute terms. Still, the regional imbalance in the supply and demand of medical personnel and the resulting medical gap are particularly severe problems, as the workforce is concentrated in the metropolitan area. The failure of the government’s policy to find the exact cause through an ongoing study of these problems and the establishment of policies to solve them is the root cause of the lack of public medical personnel. Therefore, the priority should be to identify the causes and solutions of public health vulnerabilities within the health care system, rather than to increase the quantitative and external workforce through the establishment of public medical schools.”

After the government’s announcement on July 23, 2020, negotiations took place between the KMA and the Korean government. However, the negotiations were not resolved sufficiently. On August 7, 2020, residents began a strike, and medical students stopped attending their classes. The KMA followed with a strike on August 14. Furthermore, 2,823 out of 3,172 (92.9%) applicants to the Korean Medical Licensing Examination (KMLE), including senior medical students and graduates, withdrew their applications from August 8 to 18, 2020 [6]. The government suggested holding an emergency meeting with the KMA. The negotiations, however, broke down. On August 26, 2020, the Ministry of Health and Welfare ordered physicians to start work. However, the KMA urged a more comprehensive range of strikes of physicians on the same date. There were negotiations between the KMA and the government again. On September 4, 2020, the Korean government, the ruling Democratic Party of Korea, and the KMA agreed to discuss issues on the quota of medical students and the establishment of a new medical school again from the starting point. Below is their agreement [6]:

## Policy agreement between the Ministry of Health and Welfare and the KMA

The Ministry of Health and Welfare and the KMA agree to develop a system of community medical care, essential medical care, medical education, and training for residents under the common goal of expanding the people’s health and health care system.

(1) The Ministry of Health and Welfare will stop expanding the medical school quota and promoting a public medical school. The Ministry will consult with the KMA with all possibilities open in the Medicine-Government council after the stabilization of COVID-19. Both parties will respect the results of discussions by the committee within the National Assembly, which is formed under the Democratic Party’s policy agreement with the KMA. In addition, the government will not push ahead with unilateral policy implementation, such as notification of the medical school quota.
(2) The Ministry of Health and Welfare and the KMA will form a committee on major medical issues such as the development of regional medical support measures, fostering and supporting essential medical care, substantial improvement of the training environment for residents, discussion on health care structure, and establishment of a medical delivery system. The Ministry of Health and Welfare will actively reflect and implement the results of the council’s discussions in the health care development plan.
(3) The Ministry of Health and Welfare and the medical community will discuss the development of the 4 policies raised by the KMA (quota of medical students, public medical school establishment, the pilot project for Oriental medicine benefits, and telemedicine) at the committee.
(4) To overcome the COVID-19 crisis, the Ministry of Health and Welfare and the KMA will work closely together. In particular, specific measures will be prepared and implemented to protect medical personnel and support medical institutions.
(5) The KMA will stop collective action and return physicians to the clinics.
 
September 4, 2020
Ministry of Health and Welfare–KMA

On September 7, physicians and residents agreed to stop the strike and returned to work.

## Reapplication to the licensing examination was not allowed by the Korean government and the resolution of this issue

On September 13, 2020, medical students also returned to class, and senior medical students expressed that they would like to reapply to take the KMLE. The government, however, decided that it would be difficult to give a second chance to students who previously withdrew the application, and public opinion was negative toward the attitudes of medical students.

On October 15, 2020, Dr. Yoon-Seong Lee, president of the Korea Health Personnel Licensing Examination, said the following in the National Assembly [7]:

“I understand the public sentiment that senior medical students should be deprived of the opportunity to retake the clinical practice examination as part of the medical licensing examination. The students should genuinely reflect on themselves. However, not producing physicians for such reasons can cause damage to the public. The 2 issues should be considered separately. The Korea Health Personnel Licensing Examination Institute is not authorized to decide whether or not to allow reapplication by medical students. Thorough preparations for the licensing examination can be made without a hitch following the decision of the Ministry of Health and Welfare.”

On December 31, 2020, a speaker of the Ministry of Health and Welfare said in a regular briefing that “the clinical practice exam for next year’s medical licensing examination will be conducted twice in the first and second half of the year, and the first half will be conducted at the end of January.” He added, “We will conduct next year’s examinations as soon as possible to implement measures to strengthen public health care, advance consultations with the medical community on essential medical personnel, and support vulnerable medical areas.” In his speech, the first-half exam was specially prepared for the students who withdrew their applications in August 2020 [6].

In the clinical practice examination of the KMLE taken from September 8 to November 11, 2020, passing scores were received by 365 applicants out of 423 (86.3%) who did not withdraw their application. From January 13 to 14, 2021, senior medical students applied to the 86th first-half-of-the-year clinical practice examination of the KMLE. Out of 2,709 applicants, 2,643 (97.6%) passed the practice exam from January 23 to February 18, 2021 [8]. The Korean government provided passers of the KMLE with medical licenses to be able to practice. From March 2021, they began caring for patients and people, mostly in hospitals as interns.

**Fig. 1.**
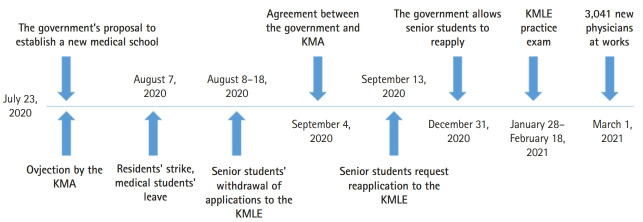
A summary timetable of key events during the period before the withdrawal of the application to the Korean Medical Licensing Examination (KMLE) by senior medical students in August 2020 to their taking the licensing examination in February 2021. KMA, Korea Medical Association.

Fig. 1 describes the course of events from students’ withdrawal of their applications on July 8, 2020 to their completion of the clinical practice examination of the KMLE on February 18, 2021 (Fig. 1). After the 3,041 new physicians received medical licenses in March 2021, the turmoil was resolved. The agreements mentioned above will continue to be discussed for the safety and health of the Korean people.

## Consequences of senior students’ withdrawal of their applications to the KMLE

A physicians’ strike is not a unique phenomenon in Korea—in fact, physicians have gone on strike in other countries to protest their governments’ policies. Of course, students’ political behavior cannot be prohibited. However, the political action of medical students related to the licensing examination was an unexpected event.

The senior medical students’ withdrawal from the KMLE in 2020 may have affected the negotiations between the KMA and the Korean government. However, if the government did not allow them to reapply for the KMLE, 2,600 graduates could not work as physicians, especially as interns at hospitals, starting in March 2021. Although it is possible to operate hospitals without interns, the recruitment of substitute personnel would be a difficult task for hospital leaders. Furthermore, existing medical personnel might experience harder work. The senior medical students probably believed that the government could not disallow their reapplication because of the emergency situation during the COVID-19 pandemic. Events happened as they wanted. I believe that those students participated in this action with sufficient knowledge and vivid goals. Some Koreans understood medical students’ action of withdrawal, while others thought it was unfair to give them the benefit of reapplication to the licensing examination. I am worried about the long-term effects of these negative attitudes towards physicians. Although their immediate goal to support the KMA’s protest against the establishment of a new medical school was successful, they probably lost a connection with or respect from many Korean people.

## Wish not to use the licensing examination as a tool for political action

Some medical policies of the Korean government may be disputable in terms of whether they provide appropriate medical care from the viewpoint of physicians and specialists in medicine and health. The issues raised by the government in July 2020 might be in such a category. However, the government may be able to continue pursuing those policies with different agendas, and the KMA and the government may work together to negotiate about these issues on an ongoing basis. If any negotiation is not resolved well, will senior medical students not take the licensing examination again? If the same action occurs next time, the Korean government may not allow medical students to re-apply for the examination, which was possible due to the COVID-19 pandemic. I hope the KMA will prevent medical students from participating in political action despite their voluntary participation. Those students’ actions made it difficult for medical schools to maintain the curriculum. Furthermore, a licensing examination should not be used as a tool for negotiation. It is a rite of passage for a medical student to become a physician. It is not too late for them to argue about political issues after becoming physicians. It should be possible for the KMA to negotiate any issues with the government without students’ supportive action. I hope that those students’ actions―not taking a licensing examination―will never be repeated, not only for themselves but also for the people.
